# Reply to Scarpati, G.; Piazza, O. Comment on “Guerrero-Romero et al. Magnesium-to-Calcium Ratio and Mortality from COVID-19. *Nutrients* 2022, *14*, 1686”

**DOI:** 10.3390/nu14163443

**Published:** 2022-08-22

**Authors:** Fernando Guerrero-Romero, Moises Mercado, Martha Rodríguez-Morán, Claudia Ramírez-Renteria, Gerardo Martínez-Aguilar, Daniel Marrero-Rodríguez, Aldo Ferreira-Hermosillo, Luis E. Simental-Mendía, Ilan Remba-Shapiro, Claudia I. Gamboa-Gómez, Alejandra Albarrán-Sánchez, Miriam L. Sanchez-García

**Affiliations:** 1 Biomedical Research Unit, Instituto Mexicano del Seguro Social, Durango 34067, Mexico; 2 Research Unit in Endocrine Diseases, Hospital de Especialidades, Centro Médico Nacional Siglo XXI, Instituto Mexicano del Seguro Social, Mexico City 06720, Mexico; 3 Department of Internal Medicine, Hospital de Especialidades, Centro Médico Nacional Siglo XXI, Instituto Mexicano del Seguro Social, Mexico City 06720, Mexico

We thank Dr. Scarpati and Dr. Piazza for their interest and comments [1] regarding our article on sMg-to-sCa ratio as a biomarker for identifying individuals at high risk of mortality from COVID-19 [2]. Dr. Scarpati and Dr. Piazza propose that iMg “…might be a better marker to identify critically ill patients with an impaired magnesium status and … (and it) may be helpful when replacing this micronutrient”. We completely agree with this statement. Magnesium is predominantly an intracellular cation and its extracellular fraction accounts only for 1% of total body concentration [3], whereas iMg constitutes 50% of total magnesemia and is electrophysiologically active [4]. Thus, the current evidence suggests that iMg is a better indicator of magnesium status. However, the measurement of both sMg and iMg is influenced by pH, changes in serum albumin (sMg), and a lack of standardization of diagnostic reference levels and selective electrodes (iMg) [3,5,6]. Thus, our main point is not whether iMg is a better indicator of body magnesium status than sMg, but to emphasize the importance of the early determination of magnesium-to-calcium balance in patients with COVID-19. The preliminary results of Dr. Scarpati and Dr. Piazza are in agreement with our report, showing that a low sMg-to-sCa ratio is associated with higher mortality in COVID-19 patients. In addition, the results of Dr. Scarpati and Dr. Piazza show that a iMg-to-iCa ratio lower than 0.55 mmol/L is associated with higher mortality in patients with severe COVID-19. Interestingly, the sensitivity of the iMg-to-iCa ratio was 0.90, whereas the sensitivity that we reported using the sMg-to-sCa ratio was 0.83. In this regard, it is necessary to emphasize that the sMg-to-sCa ratio is a measure of the imbalance between magnesium and calcium rather than a marker for magnesium or calcium deficiency. The small difference (0.07) between the sensitivities determined using the ionized and serum fractions of magnesium and calcium supports the abovementioned statement. It is important to highlight that the measurement of iMg and iCa requires specialized equipment not available in all hospitals, whereas the measurement of sMg and sCa is accessible in most laboratories. Thus, iMg is rarely measured in a clinical setting, whereas sMg is the most commonly available and employed test [3]. Finally, Dr. Scarpati and Dr. Piazza raise the following question: “does supplementation of magnesium offer a therapeutic opportunity for severe COVID-19 patients?”. We are convinced that the restoration of magnesium-to-calcium ratio imbalance might offer therapeutic advantages to critically ill patients with COVID-19. Undoubtedly, further research based on randomized clinical trials is needed to verify our hypothesis.
